# Supplementary material for: Identification of the Key miRNAs and Genes Associated with the Regulation of Non-Small Cell Lung Cancer: A Network-Based Approach
Source: Genes (Basel). 2022 Jun 29;13(7):1174. doi: 10.3390/genes13071174 (PMC9317345; doi:10.3390/genes13071174)
Supplement: Supplementary file 1 [file genes-13-01174-s001.zip › S2 File.pdf]

genes MDPI

File S2. Target genes prediction for top ranked 12 miRNAs obtained from databases (mirMap, TargetScan, miRWalk and mirDIP).

MiR	Count	Target genes (obtained from 4 databases)
MiR-30a	1076	ABL2,INO80D,THRB,UGGT1,GABRB2,ABLIM1,ZBTB34,CHD9,DSTYK,NR2F2,DEPDC1,PRRC2B,CACUL1,WWC2,ADAMTS3,FOXP2,SLAMF1,EFR3A,SOX4,RHOBTB1,HNRNPUL2,NFAT5,SNTB2,ZKSCAN2,ELOVL6,PANX1,TRIM39,SP4,RNF157,MTR,ZNF770,ZNF544,TGFBR1,MBNL2,VWA3B,PI4K2B,NEDD9,NALCN,CHD2,ATP11C,DNAJC16,CHIC1,YES1,DNAJC21,TK2,SCARB2,CPEB3,PROCR,EML4,ANKRD17,MKI67,RB1,NAA25,GMPPB,POLQ,BANP,TREML4,SORL1,CRB1,ATXN3,SYT11,ST8SIA4,NEU1,VEZT,HAUS5,DIEXF,MAGT1,FAM208A,TSPAN14,BTBD7,SOX11,LTBP1,MYC,MANEA,ARMC10,EDC3,FAXC,NCOR1,MKX,CASP3,SLITRK6,ARHGAP29,DBT,ATG16L1,PATZ1,RIF1,ROBO1,PCDHGC5,LARP6,RCBTB2,CRISPLD1,BIRC6,RALBP1,CREBBP,MAGED1,PPP1R3A,B4GALT6,CYSLTR2,TRAPPC11,TMEM87B,AP2B1,PKHD1,PNPLA8,FGD4,TBC1D9,RELN,WDR76,BACE1,LHX8,MBIP,ARV1,PIAS2,EPS8,PRKACB,FAM172A,TMEM59,SLC30A1,SLC26A2,MAP3K4,EPM2A,MORC3,LIMA1,GAB1,USP24,TMEM45B,EMB,CDK19,TBC1D23,FCRL5,EYA1,ICK,INVS,PDS5A,SPRED1,DMRT1,SYT4,NPR3,COL19A1,SS18,RAB3IP,LIN7A,TMOD2,SLC5A3,SYT2,DOK6,ANKRD12,EDEM3,FZD4,ARPP19,CDC14B,ZBTB44,CEP63,ERLIN2,CCND2,CPEB4,GATAD2B,FBXW7,RC3H1,HDAC2,KPNA6,TANC2,SLFN13,SPTLC2,ZHX1,CELF2,TRIP11,RRN3,THUMPD1,TMEM47,TET2,UBQLN1,WNK1,PAQR8,SEMA3D,NDUFA10,DDX6,TBC1D15,NEUROG2,U2SURP,ALDH9A1,DDAH1,EMP2,WDR72,HECTD2,LACC1,EID1,PDE4D,GRIA4,EIF2B5,SLC35E2B,VPS37A,RORB,EPB41L4B,UVRAG,PAG1,PRDM1,DCBLD2,XBP1,SMAD7,CACNA1D,LARP1,PDIK1L,FOXN3,USP3,UTRN,GTF2H1,SAMD8,CCT5,ABCC1,PPP3R1,RAPGEF2,RNF128,NSL1,SULF2,DLST,PLCXD3,DLGAP2,PPFIA1,RRP1B,CHCHD4,CLSPN,TFCP2,FRS2,PDE7A,TRMT10B,INTU,ALCAM,DDX18,LIN54,EOMES,FXR1,PCDHGB1,MYBL1,MSANTD4,CTR9,C11orf87,UPK1B,TULP4,PPP2R5E,PLEKHH1,FBXO25,TIGD6,NAP1L4,URB2,MAGEE1,BVES,ZFAND3,OSBPL1A,TNKS,NPPA,CSRP1,ROS1,CEP41,AKNA,IREB2,SLC30A9,TBX3,IFT20,NUPL2,CAV1,ISCU,PARP3,RAPH1,SLC5A12,SLC1A3,APOBEC3A,SKA2,PLS3,CSTF2T,PGR,SOX6,LRPPRC,MLEC,BCLAF1,FGF7,PTGR2,GOSR1,TIGIT,RPS23,NPY2R,PCDHAC1,COLEC12,GOLGA8A,UGT3A1,RRP15,EXOC8,TOMM20,DNAJA2,CNPY2,TNRC6B,SBNO1,PSD3,FOXK1,OSTM1,WASF2,FSTL4,NFIC,MIPOL1,MED13,SPAG9,EEA1,DENND5B,TMEM201,SFMBT2,GRB10,PEAK1,RHOBTB3,PREX2,PI15,FLRT3,REV3L,SLC12A7,ADAMTS5,SLC30A7,ELAVL1,LOX,CLTC,CEP97,ATP6V1A,SPCS3,SFTPB,PEX26,FGFR1OP2,FKBP5,XRRA1,URM1,TPD52,KCNC2,ZC3HAV1,SAMD12,PDZD11,ACTR8,EIF2S1,ARFGEF1,MRPL30,FGFR3,ZNF148,PRUNE2,RREB1,HS6ST2,CIT,DLG2,MECOM,DHCR24,DMXL2,CYB5RL,RNF19A,UBN2,FOCAD,NPTX1,SHROOM3,TRAPPC8,ANLN,PPP1R15B,GTPBP10,GJC1,TRIM60,CCDC62,OPHN1,FAM219A,DDX5,RALGPS2,UACA,MYT1L,NOL4,SENP2,LRRC58,SESN3,RBBP4,ADA,KDM1B,TPRG1,RHOBTB2,LRP2,VPS33A,IQCA1,NUDT11,C1S,TAB2,BEND6,NUP43,HTR7,GPR85,MOG,HMGN4,ACTR2,SYT14,TBL1XR1,SLC4A7,LIMCH1,MTAP,DNAJC2,TBC1D4,KIAA0513,FBXO8,PREPL,HOXA13,NRCAM,RALA,SLC30A4,SENP6,STRN,AKAP12,CHURC1,GABRB3,ABCC4,ANP32E,ATG12,PRPF40A,SMG1,GBP1,PRKAA2,GOLGA8B,MTHFD2,CHMP1B,SYNRG,RYR3,ZNF704,CBX5,THSD4,ERC1,PRKCA,NUS1,AFF4,MIB1,EIF4E3,GNAL,IGSF10,REEP1,ITGA2,UBA6,SMAD3,HOMER1,POM121,CD99L2,TMOD1,SNX27,DPP9,RPP30,FOXE3,EDAR,PPP3CA,ABCB9,ARHGEF12,PGP,HLA-E,PDS5B,GLCE,IL10RB,UTP15,PRR14L,GDF6,RNF114,NXPE3,HCCS,SRPRB,ILDR2,ANKRD28,SUPT16H,EPS15,CDS2,USP40,CALM1,SRA1,PTPN22,AZI2,MAPKAPK3,TAX1BP1,SCOC,CUL3,PLEKHO2,KLF11,SACM1L,NFKB1,GOLGA3,IGF2BP3,UBQLN4,SPICE1,STAT6,SLC1A2,ANXA7,CISD1,PCDHGA2,PCDHGA8,PRKAR2B,NFYA,SH3GL1,EP300,SRSF11,GCC2,SLITRK3,RNF150,ELL3,STXBP5L,NECAP2,JAG2,HECA,PRPH2,CSMD3,PUM2,MYOF,RALGAPA1,CCDC126,TMED7,ZFHX3,HSPH1,GPRASP2,DCAF12,CCDC113,ZC3H14,CCDC74B,COL24A1,INTS8,SEMA6A,PSIP1,SRP72,CD59,ZNF12,RANBP3L,MMRN1,ACTR3,SLCO1C1,KLHL15,RNF144B,EGLN1,PMEL,NHS,TRPS1,UNG,ZNF107,ESYT2,STAM2,TWSG1,TIAM1,KIAA1324L,SLC35F3,GFM1,FOXP1,MAP3K2,PCNP,KLF7,PM20D2,ZNHIT6,SP3,RAB31,PIK3AP1,ROR1,FREM2,VANGL1,PURB,PGM2L1,OTUD4,ELL2,HS2ST1,ZNF629,AFF1,FMNL3,QSOX1,TMEM245,ATRN,WDR7,SLC38A2,NFASC,ARHGAP26,FAM49A,SLC7A2,POLR3H,COL5A2,TSC22D3,MAK16,SOAT1,MACROD2,FOXK2,LIF,DRAM1,PMEPA1,PDK3,ASXL2,ABI2,P

		IGA,FOXN2,NEK9,PAFAH2,PTGES3,USP22,EME1,SNIP1,KCNA1,MBOAT2,RPS6KC1,NRAS,CD82,CTDSPL2,RARB,TTC19,ALKBH3,CRTAP,TAB3,RANBP6,VCL,DYRK2,DSCAM,MAPK1IP1L,ITSN2,PKIA,KCNQ5,MIER1,TAF2,UBE2W,ERI1,RBM45,KAZN,LRIT3,SH2D4A,RFX7,RNF138,ATAD2B,UFC1,ZNF326,EGLN3,SLC25A4,LIMD2,USP25,PCDHGA7,ADAR,SH3PXD2A,ASAP2,ANGEL2,PRRX1,HPS5,LPIN2,FAM171A1,SMG7,TMPRSS11D,RAB11B,NIPBL,KIF23,ADNP2,CRCP,PTPRD,ZBED4,PCDH18,USP6NL,SLC25A17,THSD7A,GID4,DSC3,PER3,WARS2,COL12A1,PDK4,FAM45A,ANKRD50,GABPB1,ZNRF3,EPHA4,SMAD5,WDR44,DDX17,MGA,MFSD6,NAP1L3,USP6,KLHDC1,ADAT2,ZIC4,GNPTAB,GCLM,PCDHA9,HIRA,CDC6,SIX4,NUP50,USP31,MCTP1,ANKH,UNC80,ZKSCAN1,LPP,NUCKS1,VAPB,TBC1D16,POU2F1,BNIP3L,LMAN1,LCLAT1,SNN,SPOCK1,TSN,CPEB2,TNS3,CCDC71L,PRKCQ,EEF2K,COL4A3BP,PTPN4,MED17,ACSL6,ITPKB,TOR1AIP2,MAFG,PAPSS2,LRRC8B,TGIF2,CMTM4,KIF5B,PKD2,STC1,FNBP1,ZBTB40,PARN,RPS6KB1,GREM1,IFNAR1,PCDH9,XPNPEP3,UBE2H,EZH1,RBM12,MS4A1,STC2,SYNJ2BP,HGF,ESCO1,OXSR1,PPP1R2,TMEM104,COL4A4,ACLY,TLL1,BSN,SYNPO2,RSAD1,INO80C,UXS1,ROCK1,TMEM248,TMEM237,RAB39B,ALDH1A3,SIN3A,MBTD1,POSTN,SMURF1,NFKBIE,SEPHS1,ABLIM3,PAPOLG,TAPBP,DOCK9,KITLG,CAST,RASSF8,TMEM170A,FAM13B,TDGF1,AKAP13,DLD,CCDC15,FAT4,ANKRD6,PSMC2,VEZF1,TUB,ZC3H12C,PCDHGA9,ZNF81,TRIM67,TADA2B,STAG1,GBP3,VAV3,WDFY1,PHKB,ACYP2,FNDC5,DGKE,CHD7,GPRC5B,PDP2,PSG1,AKAP9,ATP6V1C1,OTX2,SUZ12,F8,PAIP2,SST,TOP1,OPRK1,AKAP8,RCN2,NPFFR2,BDNF,ERBB4,MEAF6,F2RL2,TXNRD1,PLGLB1,SH3RF1,ELK3,FBLN7,ZNF24,AQP9,HAUS2,FLT1,MOSPD2,NUMB,CACYBP,GALNT13,FGD6,PCLO,STX6,SLC7A11,ICA1L,PLCL1,KIAA1549L,MED12L,YIPF6,FAM20B,GPR158,PCDH17,ONECUT2,TCF4,WIPF2,DCX,GLIPR1,IGF2BP1,ANKRD13C,FLRT2,TNIK,OSGIN2,POU2F2,ALDH1L2,SMAD4,ZNF260,USP38,NUAK1,SH2B3,PALM2,AKAP2,UBR3,ZFYVE26,EBF1,NUDT12,DHDDS,KANSL1L,OXNAD1,CDYL,INO80,KAT2B,PIK3R3,ZNF350,LZTFL1,RAPGEF6,CDK5R1,MFSD9,PKM,PCDHGA11,YAF2,YIPF1,BIVM,LYRM2,CNTLN,MEF2C,KLHL4,SH3BP5L,DAZL,HSPE1,SEC61A1,WAC,ATP13A3,GPR62,MSRB3,AGPAT3,ITGA5,C9orf72,TATDN3,RPP14,ZDHHC17,STX7,SEC22C,SERBP1,ARCN1,NDST1,TRIM26,XPO7,PCDHGA3,PCDHGA1,IKZF2,LATS2,OSBPL6,ASAP1,UBE2K,CLCF1,STOM,MCTP2,CASR,MAP3K5,CLDN14,PNMA2,TXLNB,SRXN1,TTC31,YAP1,DOPEY2,RNF170,LRCH1,RAD50,RGS18,MAP1B,KIAA1143,USP1,RNF41,C7orf43,BRWD1,GAD1,PHF14,HOOK1,PLA2G6,CAMK2G,ACOX1,CEP152,CAMSAP1,TAOK1,CCNG2,ATP8A1,C20orf202,DIP2B,GNS,NOX3,HELZ,SLC25A33,RNF125,ABCA1,PTPN21,FOXJ3,MPPE1,MTHFD1L,COL15A1,NUFIP1,AKT3,UBE2J1,RASSF6,RSF1,CREB1,MMP16,C1orf21,PPP1R12B,ZNF37A,LONRF2,CCDC85C,SOWAHC,CLCN4,PEG10,CBFA2T2,ZHX3,ATXN7,PTGFRN,SLC44A1,YOD1,SF3B3,CPD,MEF2D,KDM5A,TGFBR3,LMTK2,LCOR,CREG1,MYO6,FAM129B,KATNAL1,GPRIN3,HNRNPA2B1,ZNF292,OSBP,NUDT21,VCAN,FAR1,TRIM33,DDI2,ETV1,ZNF697,GRB2,RAB11FIP1,RALGAPB,BNC2,MKRN1,SATB2,MRPL19,NAA15,SLC25A43,PPP1R9A,NEDD4,MAPK6,ANKRD10,CDADC1,LGALSL,DZIP1L,CD44,GPRASP1,FRYL,CREBRF,ANKRD40,BCL2L2,IMPAD1,ZEB2,RSBN1,ARNT,ATXN1,GPSM3,FAM98A,KDM6B,USP32,B4GALT5,BDP1,SLMAP,PCDHGB7,SNX18,ZCCHC2,TCF7L1,ZDHHC7,ZNF217,FAHD1,LAP3,RHAG,GPD2,KIF4A,MARS2,LSM14A,FBXO45,SYNCRIP,CEP192,FBXO43,GAS2,TRAK1,ACTR1A,TIMM8A,VBP1,CLCC1,FSTL1,CACNB4,HOXA3,CYP7A1,SYTL2,PITX2,DONSON,DEPDC1B,MECP2,SAMD7,PDE5A,FEM1C,CDC37L1,XPO4,TRAPPC10,TUBGCP4,TBRG1,NACC2,CD109,FAM104A,GABRG1,NAPG,LCP2,DACH1,PLCB1,ZDHHC15,HSDL1,COCH,ATP10D,ARID4A,UST,NCBP1,DCLK1,UBE2G1,NPAT,NBEAL1,ANGPT2,
MiR-145	154	RAP2C,SHOC2,ATRX,DDX6,DDX3X,NCOA1,UBN1,CREB1,YES1,NUS1,SSBP2,UBR3,HBP1,TTBK2,ESRRG,RAPGEF4,LRRC2,CCDC88A,AXIN2,TSPAN12,OGT,PDZK1,RUNX1,ZNF687,ASAP1,MACROD2,EBF1,THOC2,CELF2,CNOT6L,CREB5,NFYC,R3HDM1,DLG2,MXRA7,ARHGEF12,CYP51A1,LARP4,MAPK14,TRPS1,GRIK2,HOMER2,NFIB,SOX6,AHCYL2,KCNH8,SLC1A2,PALM2-AKAP2,IREB2,MYO1B,CPQ,MPP3,MBNL2,XIRP2,RAB30,TCTN3,LPCAT3,USP33,QKI,TIAL1,TBL1XR1,PYROXD1,CELF1,KCNJ3,MCU,UBN2,SLCO1A2,LRP6,CMBL,PRKCB,NUFIP2,GARS,MBNL1,C11orf54,PAX3,ZNF423,TXNDC16,SLC5A9,ANO6,KCND2,OSBPL8,ZFX,CXCL5,CDK17,KLHL3,ARNT,SLC9A6,PIK3R3,KRT20,PTGFRN,ARRDC4,FRS2,PAX8,STAC2,ST8SIA3,DR1,COPG1,APEX1,TCF4,PTPRD,MALT1,ZFHX4,ZFYVE1,DOCK9,NELL1,RICTOR,KANSL3,GMFB,RRAS2,GDPD1,STXBP5L,FZD6,CSNK1G1,NOVA1,NBEA,PRDM2,ENTPD1,FLG2,ADAM22,RAB14,ITGB3,ADD2,ELOVL6,HOXB3,ADAMDEC1,S100A7A,METTL13,KCNK10,NR6A1,PARVA,CLCN5,BPTF,ZYG11B,TUFT1,DLX3,ASXL2,F11R,RAD52,GFI1,CTSS,NFIA,PARK7,NTN4,KHDRBS1,ZNF22,MGLL,ODF2,STAC,CMKLR1,SLC8A3,CBL,SIK2,SERPI

		NA1,SURF6,
MiR-140-3p	387	IDS,ADAM10,NFYA,PCDH17,ZNF131,GPR158,BACE1,SOBP,GOPC,NDST1,ETNK1,SGIP1,CAND1,PCDH8,STK17B,SPRED2,DNM1,ANAPC1,CBL,CCNT2,ZNF584,FGF9,TRAK1,SLAIN2,TAB2,PLEKHG3,YWHAG,PTGDR,ANKHD1-EIF4EBP3,DNAJC24,WDFY1,HIPK3,NONO,SDC4,FBXO33,LILRA2,CASD1,CHL1,SLC1A4,ZNF772,RAP2A,ZC3H6,DTX4,FANCF,CCNJ,DCUN1D1,AP4M1,GIT1,BSN,ZC3H7B,USP6,AFF4,LHFPL2,SMARCA2,BAHD1,CAPRIN1,DOCK9,ITK,SCHIP1,RAB8B,ARHGAP17,ASIC1,ATP6AP2,IREB2,IFT57,SNX25,MARCKS,ZNF594,HOXB5,HPCAL1,SIRT1,TMEM159,VANGL2,SIM1,KIAA0232,ACSS1,TRIM33,UNC5C,PPP1R3B,RAB1A,MBTPS2,DNER,HECA,POU2F1,FAM91A1,EZH1,FARP1,CEACAM6,PRDM10,USP31,CEP85,BCL2,PTCH1,CDK6,ROBO2,SLC5A3,C4orf46,QDPR,TEAD1,SOS1,E2F7,ACSM3,MAPK1,MKRN1,OTX2,GRHL2,PAK2,OTUB1,CISD1,ATP8A1,GXYLT1,B3GALNT1,SRGAP3,PHF6,CYFIP2,DGKG,VKORC1L1,RCAN3,OTUD7B,DKK2,KANSL2,SPPL3,BICD2,GOLT1B,TBX18,LYPLA1,PLEKHA1,ATF2,GRB10,ARIH1,PGR,REEP5,CHST6,MMP24,DIP2A,ITIH5,WBP1L,PHF3,EPHA8,INO80D,KCTD6,DHX9,STK38,PLCB1,GNB4,CTSD,TCF4,SLC10A4,RGS1,RIMBP2,SPIRE1,CSNK1D,NMNAT2,HS2ST1,MED13,CACNA1B,DENND5B,MEIS2,FAF2,UNC80,METTL14,PPP2R5C,HBP1,ESYT2,FAM228A,P2RY2,RNF144A,DPP8,NWD1,TRIAP1,USP38,MARK3,SUZ12,SYNC,GTF3C4,RNF44,G2E3,MTMR3,ELAVL2,NR1D2,UBXN10,C1GALT1,ZNF746,EPHA4,UBE2G2,CDK19,MRPL2,RFC5,NUDT4,SBDS,EPM2AIP1,LAMC1,MYB,RASAL1,KCMF1,KDM5A,DDC,LPGAT1,TTC21B,KCNB1,C20orf96,AGL,NBEAL1,CHMP5,HNF4G,TP53INP1,PCDH11X,EVI5,SPIN1,PARN,PDPK1,TMOD2,PRKAR2A,AFF1,CUX1,KPNA1,SAMD8,SAR1A,PAPSS2,PTBP3,ZMAT3,TCN2,NKX3-1,TNKS,MIER3,RFT1,ACER2,CYTH3,DGKD,DKK1,EIF5,MYADM,FAM120A,FSTL1,SRSF10,HLA-DOB,EGLN3,WDR36,PRKAA2,DTD1,ZNF830,C1QTNF7,CEPT1,SLC7A6,HTR1F,VGLL2,SLC22A3,LCOR,FAXC,RASSF6,ZNF516,PKIB,RNF213,TMEM245,PDK3,NFAT5,ELFN2,DDX52,RHOA,PTPN1,CHST1,ATR,DSTN,MAK,SSR3,UBE2C,PKD2,CMPK2,SRP19,LCP1,BRWD3,TLR1,RAP2B,APH1B,MAP2K6,ZNF618,MS4A14,ZNF331,NEURL1B,MTMR4,SREK1,ZXDB,HS3ST5,PLEKHG1,FOXQ1,TRPS1,TMX2,TRIM39,CDCA8,THAP6,ZNF763,MAPRE3,NRIP1,TCEA1,WIF1,ZFPM2,ZSWIM7,CMTM4,TMTC1,GJB2,TET3,C21orf91,SSR1,FAM49A,HDAC4,YKT6,ADAM17,PSMD7,SCN9A,TACC1,ZNF777,KLF5,MMP16,IFNAR1,GNRHR,C2orf83,ARID2,RTN3,SLC38A9,NMRK1,TGIF2LY,ZSCAN22,TULP4,MAFK,UNKL,MYLK4,SRP72,STX12,KIAA1147,GHRHR,SLC24A1,ZNF493,TYRO3,POSTN,HAPLN4,C9orf40,EXTL3,ZNF280B,MRFAP1,PITPNM3,FBXO48,TRPM7,PLCL1,SNAP25,UGGT1,KCTD20,BCL2L14,PHF13,HMGXB4,DCLK1,KATNAL1,HNRNPD,ENTPD4,HOXD10,NSF,PDSS1,DOCK3,AK4,ADAMTS6,WDR35,PYGB,KCNE4,UBN1,EIF5A2,KHDRBS2,PI4KB,SP4,SULF2,LUZP1,AP3S2,ADAM9,CLIC5,YAF2,CORO2A,NR3C1,SUSD5,KIF5A,FAM199X,PNPLA4,ULK4,SNX13,PAWR,RC3H1,GTF3C3,ZNF816,CD274,NAPB
MiR-572	122	PGPEP1,CREB3L2,NUDT4,RAB11FIP4,ACACA,TRAM2,PRDM2,KIAA0930,RINL,INO80D,FAM172A,WDTC1,FAM50B,KIF26B,CDH1,FCHSD1,CIB2,LETM2,CRTAM,IGHMBP2,COG3,GRIA1,KPNA6,EIF4H,ZDHHC14,ITGA11,TMEM127,MS4A7,COL7A1,PRSS36,RWDD4,PHOX2A,RAB35,ABCG5,MYPN,MTSS1L,ARHGAP12,BTBD9,WDR5,SEC14L5,PPARGC1A,SHROOM1,SLC46A1,FBXO31,MYOM3,SCAMP4,NCAM1,TMEM88,PTPRN2,CYBB,PEAK1,CD164,PPP2R2C,HARS,SPRED2,MLLT6,AFAP1,MLXIP,CMTM3,EIF2AK1,PPIB,C6orf222,PIK3R1,ATM,BAG1,NFKB1,INSM2,TAX1BP1,CD82,ARHGDIA,COL12A1,ATG9A,MVD,FAM83H,DGKG,QRICH2,EXOC7,TMC7,COQ9,SAV1,COL4A3BP,PHYHIP,DGKE,GRIN2B,TUB,ZBTB7A,QRSL1,POLR2D,ONECUT1,CLDN22,PTPRS,TRIOBP,PFKFB3,NF2,CAMSAP1,ONECUT2,HOXB6,GLIS2,MTA1,EHD1,PITPNM2,RDM1,FAM126A,NPHP3,TMC5,MMP2,SRSF3,PFAS,PCGF3,SURF4,PTPRF,CTDSP2,GRB2,SAP30BP,ARID5A,CACNA1H,MPO,SERPINH1,MRPS2,BCL2L2,GRM1,LSM14A
MiR-144	144	VEZT,CPEB3,WTAP,NR2F2,KHDRBS2,DACT3,FZD4,CDK19,MATN2,KLF9,NCKAP1,ADAMTS3,PGRMC2,DTNA,UCK2,ICK,TEK,CACNA2D1,TNPO1,SOS1,HNRNPC,LSM14B,TRIM44,SUMO3,BCL6,DCBLD2,FAM76B,FAM126B,MAFK,BMPR1A,KLF3,E2F8,HIVEP3,PPFIA1,PTCH1,PANK1,MYBL1,PDE4D,FAT3,TET2,TM9SF3,ELAVL2,SPOPL,SOCS6,H2AFV,ZEB1,ITPR1,COL11A1,ZNF507,BAZ2B,TBL1XR1,BRWD3,FOXP1,FMR1,LZIC,ELFN2,PBRM1,ZFHX3,IRS1,CNTN3,DMD,PURA,CUL3,MAP3K2,AFF4,GRM5,VANGL1,SMAD5,KCNA1,SLC19A2,CHN2,TMTC3,CTDSPL2,PCDH18,SENP7,UNC80,FAM196A,AKIRIN1,TRIM24,LBR,SEL1L,BACH2,SETD5,RARB,SNAP91,ANO1,RNF38,SMARCA4,SON,CPEB2,RAB5A,PFDN6,ESRRG,CDH8,MSI2,PYGO1,FN1,ROCK1,TJP1,QSER1,FGD6,SLC7A11,ATP8A1,BLOC1S5,HELZ,K

		IAA0232,TAOK1,C20orf194,CREB1,TBX18,UBE2D2,KHDRBS3,MITF,ASAP1,SHANK2,SALL1,ABCA1,TP53INP1,ATRX,PDCL,ROBO2,ZNF292,GABBR2,NEUROD1,SCN1A,FRYL,ZEB2,GRSF1,IKZF4,NEURL1B,UBE2G1,PPARGC1B,PTGFRN,BHLHE41,PRDM16,NACC2,GPRIN3,TTN,C3orf58,MGAT4A,RAB2A,PAPD5,MAPK6,ST18
MiR-126	11	SPRED1,PTPN9,DIP2C,PEX5,CRK,PLK2,SLC7A5,PLXNB2,LRP6,KANK2,RGS3
MiR-486-5p	99	STK35,DNAJB1,C19orf66,TMEM115,KCNJ15,SMARCD2,GXYLT1,TRHDE,TBL1X,CNKSR2,RAB11FIP4,DOCK3,NFAT5,NCKAP5,DNAJC21,BTBD3,ZZZ3,SLC12A5,HECW2,COL6A6,ST6GALNAC6,PPP3CB,DCBLD2,STK4,ZNF207,FAT3,SEMA3A,CELF2,KCNQ3,SLC4A8,ZNF507,RAP1A,RFFL,ELAVL2,PLXNA4,SCNM1,CAPRIN1,GABRB3,BCORL1,ARMC8,PLAGL2,INPP5K,KDM5B,TXLNG,IPO7,BCL11B,TMOD1,DKK2,CADM1,NFYA,PIK3R1,ACTR3,ST5,HCN1,GOLGA3,ARID4B,BTAF1,ZNF740,GRHL2,CTDSPL2,WIPF3,FGF9,MYLK2,GAB2,RASSF3,SHB,EPB41L1,FBN1,CDK4,POU2F1,TANC1,PIM1,RBM12,AFF3,GPX8,KLHDC10,CXCR5,NFE2L1,MAML2,H3F3B,REV1,SMAD2,ARHGAP44,CSPG5,SP5,FLRT2,RBM7,TMEM178B,GRIN2A,SLAIN2,C1orf21,ASB4,GPR153,UNC5C,PRRC2C,SRSF3,HAT1,IRX5,CCDC85C
MiR-210	26	KCMF1,EFNA3,E2F3,CELF2,ATG7,TET2,GPD1L,ISCU,BAZ2B,KLF7,ELFN2,MID1IP1,SYNGAP1,USP6NL,DIMT1,RAP2B,CPEB2,BDNF,FGFRL1,CYGB,ZNF462,HIF3A,NDUFA4,DTX1,B4GALT5,SCARA3
MiR-130-b	592	RYBP,NAV2,ETV3,RBM33,SYT2,UHMK1,GABRB2,MYO10,TGFBR1,VCPIP1,ZFAND5,IL6ST,RMND5A,RNF11,TNRC6C,RAB14,STOX2,SCUBE3,SPRED1,CBX6,TBC1D12,KLHL21,HLF,SLC2A1,FAM107B,DSEL,CD2AP,UGT8,BTBD7,COX7A2L,PTPRM,PRKACB,RALBP1,CHIC1,FYCO1,POU6F1,TNPO1,BIRC6,EFR3A,SLC24A3,HOXB3,MDFIC,SLC25A12,SUV39H1,PLEKHF2,BTBD3,ANKRD12,TP63,LYSMD2,NEDD4L,ROBO1,NRARP,FAM19A1,VPS13D,SEL1L3,CPEB3,CENPO,CYLD,ATXN7L1,PAX6,SRSF2,RBM17,SOX4,KCNA4,RPRD1A,SMARCD2,MAST2,F3,PSAP,CHD9,CRISPLD1,ATG16L1,NCKAP5,B4GALNT3,PTPRG,DOCK3,ATG14,UBC,MBNL1,JARID2,ATP11A,NFIA,DYNLL2,PHF20,DNAJC16,ARAP2,DEPDC1,RRAGD,MXD1,CDK19,DICER1,TSC1,MDM4,SBNO1,PPP2R5E,MIER3,ARID5B,ITGB8,PPFIA2,EPHA7,UBAP2L,MLEC,PPP6R3,FAM78A,GATAD2B,INHBA,SLC12A5,PFN2,TANC2,TACC1,TNRC6B,ST8SIA5,IGFBP5,SFMBT1,TSPYL2,ENPP6,BMP3,PDE4D,WNK1,KLF3,APPL1,LRP12,FBXO9,PDIK1L,ENPP4,RB1CC1,DCBLD2,SYT10,HIVEP2,ZPLD1,SPTY2D1,BTG1,ZDHHC23,MMGT1,CLCN6,SLC2A4RG,CPEB4,FXR1,ZNF3,NCOA1,PEX5L,AKAP1,FAT3,USP28,DCP2,SMOC1,HSPA8,SAMD8,CREB5,MTMR10,PTEN,VPS37A,ZMAT3,PIK3CB,UBE3B,SULF1,RAB9B,CDS1,DDX6,ENPP5,MYBL1,SNPH,BCL2L11,N4BP1,CSNK1G1,KCNB1,SLC43A2,ADAM12,SPOPL,RALGPS2,HECTD1,ZCCHC14,GRID2,KAT7,FERMT2,SLC12A7,TSPAN3,DLG2,HRK,TTYH3,ITGA9,LIN28A,SOCS6,DLG5,GMNC,NOVA2,PCYT1B,SOX5,RUNDC1,RAB10,CNOT6L,FIGN,EPHB4,EXOC5,GGA1,SASH1,TFDP2,NRP1,TMEM9B,SPHK2,SRSF7,PXDN,S1PR1,SLC6A6,CYP2U1,LRP2,INHBB,PPARGC1A,IL15,GRB10,PPARG,RAB34,NOL4,ITPR1,MEMO1,TGFBRAP1,FSTL5,LRRTM2,NABP1,SNX5,FOXF2,TBL1XR1,SLC35D1,ARHGAP12,CD69,RBBP8,PIK3C2A,HEG1,PRUNE2,CLTC,BRWD3,BAZ2A,TRPS1,RALGPS1,ARHGEF12,KLHL15,MOB3B,ITPRIPL2,ACER2,ELL,GRIK2,KLF6,PAFAH1B1,TEX261,FOXP1,CNOT2,ANKRD28,SCML2,TMEM151B,SNX27,PDE3B,TMOD1,MIB1,RDX,BHLHE40,QKI,MPP6,TMOD3,TOB2,HCFC2,RASGRF2,NHS,REEP1,AKIRIN2,ZNF704,HOMER1,KCTD20,ANKFY1,UBXN2B,CUL3,KIT,DIP2A,SOCS5,DIP2C,TSHZ1,AFAP1L1,NCKIPSD,VANGL1,KLF7,CCDC126,LARP4B,CAMSAP2,GMFB,THOP1,STARD13,MPPED2,TSC22D1,INSIG1,TRPC3,MLLT6,LRP1B,KLHDC8A,ACBD5,EGR3,CBFB,ARID4B,YTHDF2,BTAF1,EIF4E3,RPA2,DMRT3,FAM46B,ARHGAP1,RACGAP1,NUS1,PHF3,LAMC1,LZIC,ST8SIA3,UCP3,ERC1,CBLB,SBF2,RAP2C,POU3F2,EPS15,CFL2,SOS2,TRIM2,FMR1,ACSL4,RBM25,MAPK1,FMNL3,BACH2,AFF1,PHACTR4,TFCP2L1,SRSF10,DYRK2,VGLL3,ADCY2,ATRN,RASSF2,KLHL3,ARHGAP35,RARB,OTUD4,CCDC6,KLF13,USP46,MDGA2,MTF1,TBPL1,SLC9A2,SIX4,FUT9,IPCEF1,USP8,ZNF609,NEK9,MCTP1,NAP1L3,RFX7,ZADH2,PTPRD,CEP55,SH3PXD2A,TEX2,LBR,CEP170,CNOT7,ING1,MUM1L1,MFSD6,CDH20,THSD7A,UBE2W,PTGES3,FASTK,PMEPA1,WRN,ERBB3,NPEPL1,HPRT1,BAG5,PURG,ACVR1,KCNJ10,KDM2A,PIGA,AR,ZFP3,MAFG,FAM20B,CCDC71L,ERBB4,EPB41L1,CCND3,ZC3H12C,PLLP,QSER1,UGCG,GPCPD1,CC2D1B,RASSF3,PNRC1,ANKRD52,SATB1,TACC2,CSNK2A1,RASAL1,RAB5B,DNM2,ZNF784,E2F2,CCDC88A,ITPK1,STC1,SPOCK1,NR3C2,COG5,UNC13A,ATP2B2,CDKN1A,WDFY3,TGOLN2,TGFB2,ZNF800,TANC1,TAF4,VMP1,CPEB2,EOGT,FAM13B,TAF4B,LRP4,LDLR,FANCA,CSF1,USP33,R3HDM1,FAM13A,RAB12,MAP3K9,FIBIN,SNX2,CASD1,MED12L,TMEM63B,CPEB1,MET,RAB5A,CAST,GPR158,KBTBD8,WASL,RNF38,PTPN4,OTUD3,GJA1,WDR47,TGFBR2,MTMR9,DGKE,FBXO28,TAOK1,IGF2BP1,NAA30,CLCN5,VPS37B,RAB30,RNF213,RAB1A,HIPK3,KCNN3,AGPAT3,SERBP1,MTMR12,EFNA5,BTF3L4,BAZ1A,PRKG1,PELI1,ZDHHC2,

		KIAA1217,LNPEP,ABCA1,LRP8,STT3B,KIAA1210,RBM20,TGFA,UBE2D2,ATP13A3,MAT2B,BLCAP,FYN,INO80,STX7,UBE2D1,PITPNM2,MYT1,CCDC85A,SLC13A2,DENND1A,PHACTR2,FRMD6,E2F7,SZRD1,NHLH2,ABHD3,ZNF711,BAHD1,CCT6A,ZFYVE26,DIAPH3,SPATA2,RNF165,IRF1,LRIG1,TCF4,ARL6IP1,SHANK2,PIKFYVE,TMEM50B,LRP6,CREB1,PHF14,NUFIP2,CLOCK,BMPR2,TOM1L2,NBEAL1,LCOR,SLC39A10,DCUN1D4,ARRDC3,NDRG2,SATB2,CLCN3,PPP1R9A,CAV2,SLC44A1,SPIRE1,MLLT10,TNRC6A,ENAH,GRIN2A,SKP1,PTGFRN,ACSL1,IKZF4,IGSF3,PHF12,FAM104A,B4GALT5,SIK2,TERF2,EFNB2,HOXA5,MAPK6,LMTK2,TMEM167B,GAPVD1,AMPD3,PI4KA,ROCK2,CHMP3,TES,UHRF2,ZEB2,XPO4,CNR1,NRBF2,CMPK1,CDADC1,KIAA1468,HS3ST5,NEUROD1,CCNA2,NPAT,AKAP11,ARHGAP24,PLAA,NACC2,MB21D2,CAPRIN2,DPYSL2,ST18,PTP4A1,YY1,STX12,PLCB1,SYBU,BHLHE41,CLIP1,ZNF217,PRRG4,ESR1,VSIG10,SKIDA1
MiR-96	290	INO80D,DTNA,CPEB3,SAMD4A,PRPF38B,FRMD4A,ZBTB44,BRPF3,PDLIM5,RCAN2,UHRF1BP1,PRPF4B,CD302,NLRC5,DKK3,UBR5,ACSL3,TBC1D9,SOX11,BTRC,EMC1,HBP1,CACNA2D3,MTMR2,MAGEA10,BIRC6,ELMOD1,ANKRD12,ZNF248,MBNL1,PRKCI,ROBO1,MMD,MPP4,C1QBP,PDS5A,JMY,DICER1,GFM2,EPM2A,TRHDE,TSHZ2,LRRC4,MAN1A1,FOXO1,BCLAF1,SPTY2D1,ALCAM,KLHL28,ZPLD1,RCC2,RBM27,SCN3A,PFN2,MMGT1,LEMD3,ATP2B1,SOBP,LGR6,CYB5R4,RORB,OR11A1,PAQR8,ZMAT3,WASF3,MSTN,PACSIN1,KDM4C,KCNMB2,SKA2,TACC1,AKAP10,NIPA1,SPHKAP,RSL1D1,CCDC82,ZNF33B,REPS2,CCND2,OR51E2,CSTF2T,DNAJC6,ITGAV,CNOT6L,ELAVL2,DNM3,CAPRIN1,DENND5B,SRPK1,SLC4A10,HNRNPA3,LIMCH1,DPYSL3,RREB1,LIFR,CNTN1,MED13,FOXA1,FNBP1L,RALA,HTR7,DLL1,NFIB,FOCAD,HPS3,ITPR1,TLN2,MECOM,ZFC3H1,PDE8A,ELOVL5,ARHGEF3,H2AFV,SLC15A5,MAGEA2,TMEM200A,TMEM26,PTPN1,ST6GALNAC2,SMC2,SPCS3,GCM2,PRKAA2,TBL1XR1,GTPBP10,ZNF208,CALM1,OCLN,SSX2IP,TSHZ3,TAOK3,DLC1,CDK14,CCM2,AK5,ZNF175,PRKCA,PHF3,TRIM2,NEK2,KIAA1109,ABCB10,KHDC1,KIAA0408,FMR1,PIGF,PCNP,HCCS,STXBP5L,IFI44,CUL3,BOLL,USP47,GPR137C,PRR14L,CDS2,ORC4,WDR26,DNAJA4,FUT9,NR6A1,PTGES3,RFX7,ZNF644,DLAT,SENP7,ZNF609,CPM,GEMIN8,SLC19A2,SEC63,MYO5C,VGLL3,PAQR3,ZNRF3,WDR11,BAAT,ZIC1,UNC80,SLC7A2,SOCS4,DGKE,ETF1,CHD7,PCDH9,NR4A2,HAS2,NLGN1,WDR47,UBR1,PLCB4,ZC3H7A,HNRNPA0,WDFY3,BCKDHB,RBFOX1,PAIP2,CCDC144A,CCAR1,NUCKS1,CACNA1B,KLHL31,NAPB,ABCA5,NXT2,VAPB,ERG,METAP2,DCUN1D1,PGK1,CSPP1,LRP11,ELK4,TCF4,FLRT2,LYRM7,PRKCE,IPO5,RAPGEF6,BRWD1,KCND2,FAM107A,AKT3,TMEM92,RPS6KA3,MAP3K7,LRP8,CREBZF,YLPM1,DIP2B,UBR3,AGPAT5,REV1,USP1,YAP1,ZFY,HERC3,ARL13B,UBE4B,SETD7,TCFL5,PNRC2,STAU2,PRKG1,IQGAP2,LYRM2,CLN8,AGL,HNRNPUL1,ARHGAP44,MARK1,TMEM30A,TIAL1,TTL,GLRA2,SLC9A6,GNG2,CARD8,GNAI1,ADAMTS19,DNAJC5B,NEBL,DCLK1,PDGFRA,RALGAPB,RAD23B,PHTF2,NAA15,UBE2G1,INA,APP,MYEF2,ARID4A,NR1D2,MKRN1,SLC30A5,NDEL1,ZXDA,CYP7A1,PAPPA,HNRNPA2B1,HEY1,SLC38A1,STRAP,RSBN1,MAPK6,IL17RD,ZNF665,ERMN,ENAH
MiR-200b	573	DESI1,RNF24,HLF,CBL,CHD9,SLC31A1,FNDC3B,MBNL1,ZFAND5,ZBTB8B,ASH1L,CACNA2D1,ZNF395,ICK,CHD2,UHRF1BP1,NEDD4L,HNRNPU,SPRED1,PSME4,SWAP70,EIF5B,PKD1,KCNJ15,FAM107B,TMEM178A,FRMD4A,ARIH2,TBX5,SORT1,CDH11,KCMF1,ZFHX4,DR1,NXPH1,PTPN13,FNBP4,DTNA,NDN,TMCC1,SIAH1,HDHD2,VCPIP1,SDK2,POU6F1,KDELC1,MXD3,MOB4,E2F3,SCD,AMOTL2,ADAMTS3,MYLK,RELN,PCDH19,NCOA2,GAB1,SMAD9,KIAA0895,GPR107,YWHAG,ATP11C,EPS8,PSAT1,PRKACB,N4BP2,C16orf72,SNTB2,SEC23A,GNAQ,NFIA,SLC5A3,ZC3H6,SOX6,KRT80,CELF2,SCN2A,MIER3,GATAD2B,KRAS,HNF1B,FGF18,PTCH1,ARID5B,TBP,RAPGEF1,GREM2,NPNT,PFN2,HIVEP3,CASKIN1,VAT1L,PRDM1,TRIM44,FBXL17,HMBOX1,EFNA1,PLS3,PIK3CB,ELF2,MBD5,CDKN1B,RAB37,RAPGEF2,SMARCAD1,ANO6,NANOS1,NOG,PCSK2,FXR1,LIN7B,PISD,CCDC82,PPP6R3,SLC6A11,FBXW7,ZMAT3,CEP41,SAMD8,ETS1,PPP2R5E,CREB5,ADIPOR2,CNN3,CASC4,PLCXD3,TLL2,CCNJ,PIGM,WDR91,KRR1,GPM6A,ZNF532,FAT3,RECK,WASF3,DCBLD2,ARL5A,TBL1XR1,SESN3,ZNF148,RAB3B,GABRB3,HIPK2,RLIM,CEP97,SOX5,ZCCHC14,SLC4A4,FHOD1,NFIC,PAPLN,PDE7B,RABEP1,ANGPTL3,KDM3B,PDPK1,WASF1,RLF,APLP2,UBN2,ZNF236,NFIB,SCN5A,SMARCD1,ZNF365,NOVA2,PSPH,ARL3,SDC2,TLN2,PLXNA4,SMURF2,KCNB1,BAZ2B,PEAK1,FAM219A,HEG1,CASZ1,RDH10,BASP1,PCDH8,MAPRE1,LEPROTL1,ANLN,KDM1B,GRID2,CITED2,SPAG9,SENP5,ITPR1,SLC4A7,LPIN1,FOXK1,DHX36,GATA4,BRWD3,FUBP3,TRAPPC8,NRIP1,EIF2S1,TFAP2A,RALGPS2,ARHGAP19,MCFD2,MED13,NLGN4X,C6orf120,SECISBP2L,ZYG11B,STRN,ELAVL2,LOX,FIGN,ZEB1,ST8SIA3,FREM2,ACTR3,ERC1,DFFA,RAB7A,ELL,CALHM1,AFF4,FOXP1,MPP5,SCN3B,HDAC4,ACVR2A,SLC38A4,FARP1,DMD,PRKCA,CBX5,NPC1,KLF6,BCL9,SAR1B,IGF2R,SLC2A14,FRMD4B,EXOC6B,SH3GL1,SLC16A2,BCL11B,APAF1,AUTS2,PHF6,NCOR2,EP300,GATA2,PRKAR

		1A,FMR1,CUL5,KANK1,SYDE1,NEO1,CHSY1,TSC22D1,YWHAB,PPFIBP1,TRIM2,CHSY3,SGIP1,ABAT,HIPK1,SRP72,PRKAR2B,RASA2,ST3GAL2,STARD13,HCFC2,WWC3,INTS8,PDS5B,PCNP,TMEM189-UBE2V1,TMEM189,FUBP1,TAOK3,S100PBP,CKAP4,EIF4E3,LPAR1,ANKRD28,RAB18,LAMC1,NOVA1,RAP2C,CAMSAP2,LRP1B,ZFPM2,GMFB,ANO5,TWISTNB,FUT4,TMOD3,QKI,SLC1A2,DYRK2,GNA13,AFF1,RFX7,KLHL3,ASXL3,BACH2,PKIA,TMEM229B,SBF1,MGA,CNST,NKD1,CCNYL1,JAKMIP3,TEC,CLIP2,NIPBL,ADCY2,SLK,ANKH,TAF12,ANGEL2,BICD2,CORO1C,DNAJC18,CLASP2,CTNND2,SGCE,USP25,LPIN2,TMEM245,NEK9,CHD1,UBE2W,MAPK1IP1L,ZNF423,DPY19L1,DENND5A,SRSF10,CDK13,PTPN12,USP6NL,RHOA,CRTAP,BAG5,GAL3ST1,PAQR3,UBE2V1,CSRNP3,MSN,CECR2,OTUD4,RND3,AP1S2,ERI1,MBOAT2,ABI2,HS2ST1,CYP1B1,NR5A2,POLK,USP31,DCAF17,FAM8A1,CEP85L,SLC6A17,ERBB4,CCDC117,MLLT3,TMEM237,RBFOX1,RNF38,TJP1,ERG,TUBB,ETF1,ELK3,SEPHS1,PKP1,AMBRA1,SLC24A4,NRBP1,NUMB,PAIP2,RTF1,AEBP2,NR2C2,ZCCHC24,ZBTB5,DAG1,ATP2A2,SRF,ING2,CPT1A,TP73,HAS2,KIAA0355,FLT1,PAPOLG,SPATS2L,SIN3A,LRRTM3,PHLDB1,TMEM170A,AFF3,STXBP6,TAF4,PLCL1,MED6,TRIO,PMPCB,ESRRG,KLF10,NAPB,ZBTB7C,GRIP1,LRP4,OSMR,GAN,TMEM17,UBE2B,KLF12,VASH1,RBFOX3,FN1,UGCG,NRG1,TMEM33,DIRAS2,MBLAC2,ASF1A,FBXO22,MOSPD2,RPS6KB1,ZFX,RASSF8,TAOK1,TCF4,ONECUT2,CREB1,ABCC9,ZC3H4,AKT2,C7orf43,CGGBP1,SRGAP1,SETD7,UBE2J1,PRKG1,GNAI3,BTF3L4,BAP1,ABCA1,MXI1,ESRP1,FAM126A,FBXW2,COPS8,FAM168B,SRSF1,RBM20,ATL2,ASAP1,BRWD1,ELAVL4,LATS2,CASR,WDFY2,MMP16,SLITRK1,ATMIN,RUSC2,CEP350,GLCCI1,ZNF711,SFXN1,CHST2,KANK2,DUSP1,FOSL2,NCS1,CNOT4,SCHIP1,SEMA3F,ORMDL3,LRIG1,MAP3K5,PPP6C,IER5,NUP153,ITSN1,MTFR1,TIMP2,MYT1,INPP4A,TIAL1,RANBP10,CDH6,MAP1B,PPM1F,ZNF652,SLIT2,ARIH1,PTPN21,HOOK1,ZDHHC17,ATP11B,MAP2,STYX,HIPK3,ELK4,NUFIP2,MEF2D,USH2A,IDS,GPRIN3,WDR82,PI4KB,UBXN7,NACC2,PTP4A1,SCN1A,ZNF280C,ROBO2,CACHD1,WBP1L,SIX1,SLC23A2,MYO9A,FGF12,PIP4K2A,ACTR1A,GALNT2,VLDLR,ARID4A,MSL2,ANK3,IGSF3,SCRT2,IMPAD1,TMEM136,GBX2,ARHGAP20,NR3C1,RAB23,BHLHE41,GIT2,BAG6,SLC30A5,GRIN2A,SLC6A1,NEDD1,CLIP1,DNMT3B,NBR1,LMO7,DNAJB14,ROCK2,NEGR1,PAPD5,ZNF292,CDK17,BNC2,CERS6,MARCKS,PPP1R12B,WNK3,LCA5,CLOCK,WIPF1,BDP1,TRIM33,RAB11FIP2
MiR-205	832	TNPO1,FOXP2,SOX11,PLAG1,MAPK8,PFKFB2,RYBP,PDPR,FAM102B,PLA2R1,ZFP90,DNAJC21,SYT6,FAM172A,FRMD4A,NCKAP5,SP4,SLC27A6,SS18L1,SORL1,MAP7D2,EMB,AP2B1,COL19A1,SNX11,TMEM45B,TPK1,SLITRK6,CPEB3,MBNL2,NCOR1,CTTNBP2NL,POT1,FAXC,TMEM38B,ARL4A,DSEL,KCNA4,OLFM3,ZC3H6,TCF12,PNPLA8,ABCA12,TMF1,RTN4,C5orf30,PKN2,VEZT,CDH11,TRAPPC11,FAM208A,VASH2,DTNA,CYSLTR2,ZNF395,HECW1,IL6ST,GFOD1,TMCC1,RNF103,DUSP22,RIF1,EDEM1,CHCHD1,CDK19,ERMP1,GEMIN2,RGPD5,LIMA1,POU4F1,GLO1,CWC27,MORC3,TMEM59,TNFAIP6,AFTPH,PRKAG2,NAV2,SYT4,ZBTB10,F2RL1,FAM19A1,SLC35D3,N4BP2,PRDX3,ST8SIA4,PPP1R3B,C3orf70,MDM4,PTPDC1,SLC30A1,PCDHA8,PCDHAC2,NFIA,JAZF1,EDEM3,SPRED1,SWAP70,SREK1IP1,CEP63,TOPBP1,CCNT2,MEGF9,LEPROT,FAM199X,ARPP19,VCPIP1,ICK,DOK6,XIRP2,CA8,RBMS1,ANKRD12,CHML,ZBTB44,RC3H1,GOSR1,ETS1,LARP1,TNRC6B,ARID5B,PAG1,ARID2,WNT5A,MIER3,HBS1L,SOS1,DCAF5,BCLAF1,ZFAND3,ATP2B1,TET2,VTI1A,WNK1,URB1,PANK1,RAB28,FXR1,LRPPRC,TRDN,PDE7A,ZSCAN30,RAPGEF2,MSMO1,VPS37A,SLC17A5,IREB2,RAB37,PGR,PRDM1,MS4A7,STK24,CEACAM6,PTEN,HNRNPR,RAB6A,WDR72,ZNF682,TAF9B,DDHD2,SLC35G3,EPB41L4B,C5,KCNH8,NCOA1,ZHX1,KDM4C,CEP41,ADCY9,ERCC6L2,NPTN,FAM122B,INTU,MYO5A,SPHKAP,ISCU,ADNP,FBXL17,HSPA12A,IRS2,PARP3,STXBP4,DLGAP2,LTB4R,SESTD1,CELF2,PPP2R5E,SPTBN1,NSRP1,GPC5,TEDDM1,ZBTB43,ZNF576,TXNDC16,GIPC2,ELF2,RECK,LRIF1,TBCK,NSL1,IKBIP,NANOS1,FAM216B,PCDHAC1,PCDHA10,CDS1,TOMM20,LGI2,SAMD8,SEMA3A,SLC1A1,RXFP1,CAPZA2,SEMA3E,ATG4C,GPR180,TNKS,MSANTD4,MKLN1,STEAP2,EXOC8,UGT3A1,PLCXD3,CNOT6L,WASF2,DENND5B,SPIN1,TBL1XR1,ATP2B4,FNBP1L,TRIM25,SOX5,WDR37,NOL4,TLK1,PKNOX2,TRIB2,NRCAM,COBL,SYT14,DLG2,C2orf88,SELE,LIMCH1,MAPRE1,MYCBP2,KIAA0513,ANP32A,SCAF11,ZSWIM6,UACA,GABRA4,NCAM1,ST8SIA1,ARHGAP12,STX3,ZNF148,TRAPPC8,RBBP4,REEP5,TFDP2,SASH1,UBE2E1,KLK10,SRRM1,THOC2,MECOM,CPPED1,SLC16A6,TP53AIP1,FBXO8,UHRF1BP1L,RGPD6,GABRB3,DERA,CCPG1,HTR2C,MIPOL1,ATG12,NFATC3,SDK1,PTTG1IP,MNT,FGF5,METTL21A,SENP2,KDELC2,PREPL,TMEM54,AKAP12,ABCC4,PCDHA11,FREM1,GPR85,RICTOR,XKR9,LUZP2,HIPK2,RYR3,ARHGAP19,MTHFD2,HOXA13,ANP32E,LPGAT1,SMG1,SEMA3C,SYNRG,CSGALNACT2,SMCHD1,SESN3,DNM3,SLC4A7,MAP3K2,ST8SIA3,FMN1,STXBP5L,ZFHX3,TRPS1,RFX3,TRIM2,SLC1A2,PAFAH1B1,BCL11B,STK38,ANKFY1,PIK3R1,NHS,LIX1,PRR14L,AQP4,SPOCK2,FAS,

	TSHZ1,EXTL2,CDK12,HMGB3,VPS36,INTS6,UNG,GOLGA3,MPP6,LAMA4,TNFSF11,TOX3,MAP2K4,CDC42SE2,PUM2,SMARCA1,PRKAR1A,SGIP1,PIK3AP1,DMRT3,HEXIM1,ETFA,SEMA6A,RALGAPA1,ANKRD20A1,TRNT1,IL22RA2,DENND4A,ARID4B,PURA,PNPT1,TSC22D1,AP1S1,RAB27B,OR2H1,ITCH,GFM1,ANKRD28,EVI5,DUS4L,SCN3B,FOXP1,PMEL,COL3A1,RANBP2,OSBPL3,JRKL,GCLC,DST,PLS1,WWC3,PARVA,ESYT2,MALT1,VANGL1,KLF7,HOMER1,PCDHA1,PCDHA4,ARHGEF12,ADAM10,SRSF11,LMO3,ARL1,GMFB,SSX2IP,MPP7,FREM2,FKTN,APC,FGF2,LPP,PURB,SOCS4,CSRNP3,DSC3,NR5A2,ZNF641,BACH2,RAP2A,SIX4,ITSN2,MACF1,KCTD12,MUM1L1,MYO5C,KIAA0319,NIPBL,MTF1,CEP170,TARDBP,GPBP1,KCNE4,EPHA4,CLDN2,PIGA,CCDC25,NCOA6,SLC7A2,WDR44,PTER,CLINT1,TMC7,FAM196A,MFAP3,CPM,UBE2V1,HPS5,METTL9,KCNQ5,IFT57,GID4,TMPO,CD19,RTCA,GCLM,MED13L,TIA1,NUDT15,UBE4A,ZIC4,C11orf53,THSD7A,ZBTB33,CALD1,SH2D1B,HIRA,CLCA4,SSR1,MAF,GNA13,TFCP2L1,ASXL3,NIPSNAP3A,ARMC1,ANKRD50,SLC38A2,USP6,CDK13,RASSF2,CEP68,HS2ST1,ZDHHC21,PSMA5,GJA5,FAM35A,MGA,GNPTAB,FAM49A,PCDHA5,PCDHA9,PCDHA7,PKIA,ZBED4,ATAD2B,TRMT5,PDK4,ZNF644,TTC30A,DLAT,MTX3,UNC80,ZKSCAN1,ARL5B,SV2B,RAB22A,ERBB4,TMEM237,CPEB2,KLHL31,PDE11A,ELK3,UBE2H,SPOCK1,PCDH9,OSBPL11,YWHAE,HMGA2,NCOA7,NR3C2,AP4E1,RBM24,MBTD1,RBPJ,ATP6V1C1,CELF1,NRF1,RHOQ,ARFGEF2,PIK3C2B,KLF12,KIAA1549L,GNA12,RNF38,CSDE1,CCDC71L,IPO8,CCND3,CDHR3,RAPGEF4,ANKRD6,RPS6KA6,NDFIP1,ZCCHC6,RBBP5,RHOT1,HMGN2,CHD7,RBM19,GRM7,SHISA5,MCM10,TOP1,VEZF1,MGAT3,SP1,CRNKL1,XPO1,LRRC8B,LINGO1,PLCL1,SNX25,CLDN22,LRRTM3,COL4A3BP,BDNF,PUS7,USP33,GPC4,LHX6,GABRP,NT5DC1,NVL,GPX8,ELF1,ACYP2,RASSF8,TXNRD1,DLD,TMCO3,TARSL2,AKAP9,DUSP16,PTPRC,CCDC15,ITPK1,DARS2,NHSL1,DRAM2,RPS6KB1,ENOPH1,VAV3,EPG5,SNX14,PYROXD1,COL4A3,TMEM33,UBE2R2,ZNF24,PSG1,ABCA5,MS4A1,KCNJ13,CBLN1,ASB14,ZC3H12C,PCDHA13,PCDHA12,SUZ12,GPR158,KMO,DTL,CPED1,PCDH17,GPCPD1,ANO3,CCDC88A,ICA1L,TRDMT1,FAM20B,NAA50,YIPF6,NLGN1,ONECUT2,NAV1,DCX,HELZ,FAM107A,IKZF2,YWHAZ,SEMA4F,ADAM28,MEF2C,AKT3,MAP1A,SETD7,EDA,E2F7,SFRP1,ASAP1,EBF1,BAHD1,WIPF2,YAF2,DNAJC11,ABHD2,CGGBP1,SLIT2,UBR3,TRMT1L,MAP3K7,CTSO,NRK,FBXW2,CAP1,CHMP2B,SLC16A7,ZDHHC17,EIF2B1,CNOT4,NAIP,RAD21,ZNF596,TBC1D8B,PHF14,LRCH1,PPP6C,CAB39,MPV17L,RRM1,TCF4,HSPE1,YTHDF1,DZANK1,CALCR,ADH7,SLC25A44,CSPG5,PLA2G6,CDH6,MAGEB4,MAP3K5,WAC,JAM3,BAMBI,SMAD2,KIAA1143,MCTP2,ADAP2,CCT6A,AGPAT3,FAM135A,NEUROD4,SIRT1,SLCO1A2,TRERF1,NOX3,MRC1,ASPH,DYNLT1,NUDT12,PCDHA6,PCDHA3,RASSF6,CREBZF,PAH,BRWD1,NUFIP1,SFXN1,AGPS,C5orf63,VPS13A,TMEM30A,CEP350,ENTPD1,CDK6,MMP16,NEBL,NUFIP2,CACNB4,RAB11FIP1,SLC23A2,PPP1R9A,SEMA5A,ZNF664,PPP1R12B,OSBP,GRSF1,LCOR,DCLK1,DAB2,ST18,ARF3,PJA2,TGFBR3,SORBS1,GRIA3,USP12,TNFAIP8,IL17RD,ITGA6,CHMP4B,TMEM167B,PAPD5,SMAP2,MLLT10,KDM6B,RBM22,RNF141,KIAA1549,ME2,TP53INP1,PI4KB,MYEF2,SAP30BP,CDC37L1,HMGCS1,PHTF2,UNC13C,MEST,DDX58,CD28,PCYOX1,VKORC1L1,WIPF1,GPD2,GFRA1,FAM98A,DDX42,SNX18,CHL1,VBP1,SLMAP,FZD6,COCH,ENOX2,SHISA9,GLRB,SUSD5,DONSON,CD44,CPNE4,TIMM8A,CEP192,ITGA1,SATB2,LRRCC1,PCDH11X,SLC38A1,ABI1,MECP2,NOD2,SPIRE1,ADAM7,ATXN1,ZNF292,DEPDC1B,ZDHHC15,LCA5,NSD1,MCMBP,CDK17,SLC39A10,THEMIS,NBEA,AZIN1,RAB3GAP2,GPRIN3,UNC5C,ZNF772,PLCB1,SLC12A1,TRUB1,USH2A,CAND1,LYPLA1,FAM104A,CPD,RAB11FIP2,ARID4A,NNT,MAOA,ANGPT2,ATP10D,TET1,USP13,RSBN1,ATP8B4,DDX3X,CLOCK,YOD1,KIAA1468,ATRX,NBEAL1,TMEM178B,FEM1C,RAB8B,SLC12A2,IMPAD1,GABRG1
