# Supplementary material for: Identification of the Key miRNAs and Genes Associated with the Regulation of Non-Small Cell Lung Cancer: A Network-Based Approach
Source: Genes (Basel). 2022 Jun 29;13(7):1174. doi: 10.3390/genes13071174 (PMC9317345; doi:10.3390/genes13071174)
Supplement: Supplementary file 1 [file genes-13-01174-s001.zip › S3 File.pdf]

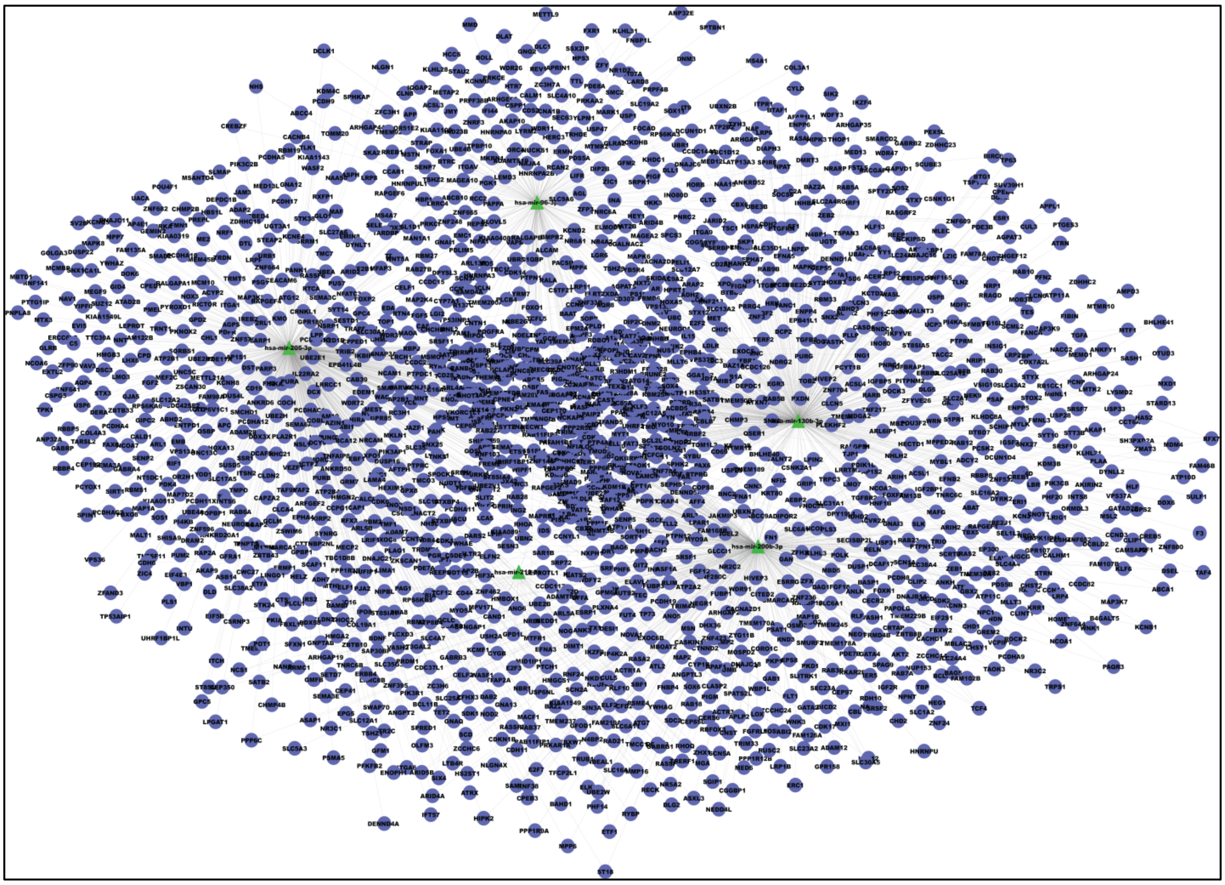

Figure S3. MiRNA-target gene interaction network. The target genes were obtained from different databases and the common ones proceeded forward. The miRNA-mRNA network contains 1728 nodes and 1928 edges that were constructed using Cytoscape software. Triangle (green) represents Upregulated miRNAs and circle (blue) represents the interacting gene partners.
